# Retraction Note: Long noncoding RNA HEIH depletion depresses esophageal carcinoma cell progression by upregulating microRNA-185 and downregulating KLK5

**DOI:** 10.1038/s41419-023-06365-z

**Published:** 2023-12-18

**Authors:** Bing Wang, Xuezhi Hao, Xingkai Li, Yicheng Liang, Fang Li, Kun Yang, Hengqi Chen, Fang Lv, Yushun Gao

**Affiliations:** 1 Department of Thoracic Surgery, National Cancer Center/National Clinical Research Center for Cancer/Cancer Hospital, Chinese Academy of Medical Sciences and Peking Union Medical College, 100021 Beijing, China; 2 Department of Medical Oncology, National Cancer Center/National Clinical Research Center for Cancer/Cancer Hospital, Chinese Academy of Medical Sciences and Peking Union Medical College, 100021 Beijing, China

Retraction to: *Cell Death and Disease* (2020) 11:1002 10.1038/s41419-020-03170-w, published online 22 November 2020

The authors have retracted this article because they have found that the results are not reproducible. An internal investigation of the experimental records has found that there were errors in the original data, as the analyses were performed on a single replicate.

Additionally, concerns have been raised regarding high similarity of western blot and Transwell assay images between this article and a number of other articles on similar topics published within a similar time frame. The authors have been unable to provide the full gel images of the western blots, and have stated that the Transwell experiments were outsourced to a third party.

All authors agree to this retraction.

